# Effects of Tick-Control Interventions on Tick Abundance, Human Encounters with Ticks, and Incidence of Tickborne Diseases in Residential Neighborhoods, New York, USA

**DOI:** 10.3201/eid2805.211146

**Published:** 2022-05

**Authors:** Felicia Keesing, Stacy Mowry, William Bremer, Shannon Duerr, Andrew S. Evans, Ilya R. Fischhoff, Alison F. Hinckley, Sarah A. Hook, Fiona Keating, Jennifer Pendleton, Ashley Pfister, Marissa Teator, Richard S. Ostfeld

**Affiliations:** Bard College, Annandale, New York, USA (F. Keesing);; Cary Institute of Ecosystem Studies, Millbrook, New York, USA (S. Mowry, W. Bremer, S. Duerr, I.R. Fischhoff, F. Keating, J. Pendleton, A. Pfister, M. Teator, R.S. Ostfeld);; Dutchess County Department of Behavioral and Community Health, Poughkeepsie, New York, USA (A.S. Evans Jr.);; Centers for Disease Control and Prevention, Fort Collins, Colorado, USA (A.F. Hinckley, S.A. Hook)

**Keywords:** tickborne disease, Lyme disease, ticks, *Ixodes scapularis*, prevention, vector-borne infections, New York, United States, zoonoses, bacteria

## Abstract

Tickborne diseases (TBDs) such as Lyme disease result in ≈500,000 diagnoses annually in the United States. Various methods can reduce the abundance of ticks at small spatial scales, but whether these methods lower incidence of TBDs is poorly understood. We conducted a randomized, replicated, fully crossed, placebo-controlled, masked experiment to test whether 2 environmentally safe interventions, the Tick Control System (TCS) and Met52 fungal spray, used separately or together, affected risk for and incidence of TBDs in humans and pets in 24 residential neighborhoods. All participating properties in a neighborhood received the same treatment. TCS was associated with fewer questing ticks and fewer ticks feeding on rodents. The interventions did not result in a significant difference in incidence of human TBDs but did significantly reduce incidence in pets. Our study is consistent with previous evidence suggesting that reducing tick abundance in residential areas might not reduce incidence of TBDs in humans.

Lyme disease is an emerging zoonosis caused by the spirochete bacterium *Borrelia burgdorferi*, which is transmitted between vertebrate hosts, including humans, by ticks in the *Ixodes ricinus* complex. Annual cases of Lyme disease in the United States, as reported to the Centers for Disease Control and Prevention (*1*), have grown from a few hundred in the early 1980s to >30,000 in recent years. A recent study estimated that actual clinician diagnoses of Lyme disease in the past decade exceed 450,000 per year (*2*,*3*). Increasing incidence over the past few decades reflects both upward trends in case numbers within Lyme disease–endemic locations and a dramatic geographic spread from both northeastern and Midwestern foci (*4*–*6*). Beyond the effects of Lyme disease on human health, economic costs of patient care are estimated at ≈$1 billion/year in the United States (*7*).

Preventing exposure to *B. burgdorferi* and other tickborne pathogens can be aided by personal practices such as applying repellents, checking for ticks, and avoiding tick habitats. However, the efficacy of these methods is unclear, and considerable differences in effects have been reported (*8*,*9*). Although specific methods of property and wildlife management (e.g., deer hunting) are advocated by some agencies (*10*), knowledge of the effectiveness of these recommendations in reducing human encounters with ticks and incidence of tickborne diseases (TBDs) is limited (*11*–*13*).

Controlling the size of tick populations is generally considered a promising way of reducing human exposure to TBDs. Researchers pursuing these methods have identified chemical and biological agents, including synthetic pyrethroids, organophosphates, and entomopathogenic fungi, that are lethal to ticks (*14*–*19*). Field trials generally show that application of chemical or biologic acaricides can reduce the number of ticks by 50%–90% (*20*–*22*). Combining acaricides with other interventions (e.g., wildlife and landscape management) has also been assessed. However, studies evaluating whether these integrated approaches reduce human exposure to ticks are limited by design constraints, such as the lack of masking of researchers to treatment assignments, lack of appropriate placebo controls, small scale of deployment, unbalanced designs, and low statistical power. Studies also do not generally include data on human health outcomes, particularly incidence of TBDs (*23*,*24*).

A recent study (*23*) rectified many of these deficiencies by applying an acaricide (bifenthrin) to 2,727 residential properties in 3 states; using a masked, placebo-controlled design; and including tick abundance, human encounters with ticks, and cases of TBDs as response variables. Despite showing >60% reduction in tick populations on properties treated with the acaricide versus the placebo control (water), the study (*23*) showed no reduction in either tick encounters or cases of TBDs. One potential reason for this lack of effect is that the treatments did not reduce tick abundance below some putative threshold needed for reduced disease risk. A second possibility is that humans might frequently encounter ticks in locations other than their yards. In both cases, tick control might be more effective at reducing tick exposures when applied throughout a residential neighborhood.

This study, the Tick Project (*25*), was designed to determine whether tick control, when implemented more broadly in residential neighborhoods and by using multiple approaches to tick management, could reduce TBD risk and incidence. We designed a randomized, replicated, fully crossed, placebo-controlled, masked experiment to evaluate whether 2 environmentally safe methods to manage ticks, used separately or together, reduced tick abundance, human and pet encounters with ticks, and human and pet cases of TBDs.

## Methods

We tested the effects of 2 methods of tick control, used separately or together, on tick abundance, tick encounters with humans and pets, and cases of TBDs over 4 years (2017–2020) in 24 neighborhoods in Dutchess County, New York, USA. The first intervention, the Tick Control System (TCS) (Select TCS, Tick Box Technology Corporation, http://www.tickboxtcs.com), consists of baited boxes that attract the small mammal hosts most likely to infect ticks with pathogens. When inside the box, these mammals are brushed with a dose of the acaricide fipronil. The second intervention, Met52 (Novozymes Biologicals, https://biosolutions.novozymes.com), is a fungal spray developed to kill questing ticks. Both interventions have been demonstrated to have extremely low toxicity to humans, pets, and wildlife as applied (*21*); high specificity for ticks (*26*); evidence of efficacy in tick-control as revealed in small-scale studies (*15*,*20*–*22*,*27*); and commercial availability at the time of the study.

The design was fully crossed so that 4 treatments were used: placebo TCS boxes and placebo Met52, placebo TCS boxes and active Met52, active TCS boxes and placebo Met52, and active TCS boxes and active Met52. All participating properties within a neighborhood received the same treatment. We included 6 replicate neighborhoods in each of 4 treatment categories to achieve 80% power to detect an effect size of 60%. Given the intensity of treatments and length of the study, increasing the sample size to achieve greater power was infeasible. Selected neighborhoods had high incidence of Lyme disease and moderate to high density of 1- and 2-family residences. During April 2016–June 2017, residents were recruited by mail, telephone, and in-person visits. Neighborhood treatments were randomly assigned, and study participants and scientific personnel that collected or managed data on response variables were masked to treatment assignments (Appendix). 

Beginning in spring 2017, we deployed the 4 treatment combinations on participating properties (Appendix Table 1). We deployed TCS boxes or placebo boxes that contained no acaricide at densities consistent with product labeling during spring and summer, corresponding to the activity peaks for nymphal and larval blacklegged ticks (*28*). We placed boxes >10 meters apart in all habitat types that we sampled for ticks and placed them in protected locations, such as along building foundations and under vegetation, that are frequently used by small mammals.

If effective, TCS bait boxes would kill larval (hatchling stage) ticks feeding on small-mammal hosts in summer and fall, leading to fewer nymphs (second immature stage) the following spring. Met52 would kill questing nymphal ticks in spring. Our tick sampling focused on the abundance of questing nymphal ticks in spring and ticks on small mammals in summer.

Met52, which contains spores of the F52 strain of the entomopathogenic fungus *Metarhizium brunneum*, was prepared according to product label instructions and applied by using truck-mounted high-pressure sprayers. Identical trucks and sprayers were filled with water for the placebo controls. Spraying was conducted twice each year preceding and during the peak of activity of questing nymphal ticks (*28*). For properties that included extensive forested areas, spraying extended 12 meters into the forest.

During the peak activity period for questing nymphal ticks and at least 1 week after spraying, we used 1-m × 1-m white corduroy cloth to flag-sample ticks at 20 randomly chosen participating properties within each neighborhood, sampling 3 habitat types on each property: lawn, forest, and shrub or garden, whenever present. To assess tick burdens on small mammals, we conducted mark-recapture sampling by using Sherman live traps at 10 participating properties in each neighborhood during August and September 2017–2019, corresponding to the activity peak of the larval stage (*28*). We did not conduct sampling in 2020 because of the coronavirus disease pandemic.

In an introductory survey, we asked the primary contact for each household where and how frequently each member of the household spent time outdoors and what approaches to personal tick prevention they used. From spring through late fall each year (Appendix Table 2), we distributed biweekly surveys to each participating household, asking whether any full-time resident, including pets that spent time outdoors, had encountered a tick or had a TBD diagnosed in the previous 2 weeks. We asked participants who reported TBD in humans to consider signing a medical consent form to enable confirmation of the case by their healthcare provider.

We generally evaluated effects of treatments by analyzing data aggregated at the neighborhood level to determine the effects of each treatment alone and in combination (Appendix). For tick encounters and cases of TBDs for humans and pets, we accounted for numbers of participants within neighborhoods. The Institutional Review Board and the Institutional Animal Care and Use Committee of the Cary Institute of Ecosystem Studies (Millbrook, NY, USA) approved protocols involving informed consent by human participants and the live-trapping and handling of small mammals.

## Results

### Characteristics of Neighborhoods and Participants

The average neighborhood was 27.5 (range 12.9–39.2) hectares and contained 118 (range 77–162) properties; average parcel size was (range 0.02–1.8) 0.19 hectares. A mean of 43% (range 18%–63%) of the neighborhood consisted of forested habitat, whereas lawns, shrubs, and gardens together accounted for ≈30% (range 14%–48%).

**Figure 1:**
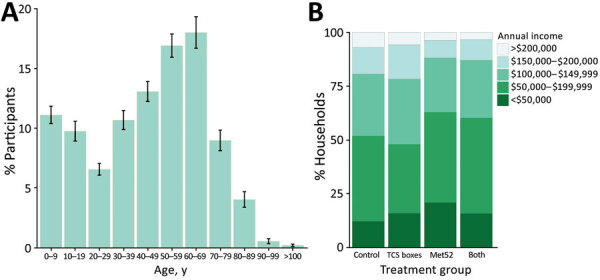
Characteristics of participants in study of tick-control interventions in residential neighborhoods, New York, USA. A) Mean percentage of participants in each age category at the time of enrollment, averaged for 24 neighborhoods. Error bars represent SEM. B) Mean percentage of households in each category of annual household income, averaged for the 6 neighborhoods in each treatment group. TCS, Tick Control System.

During the recruitment phase, ≈25% of households in each neighborhood did not respond to repeated attempts at contact, ≈25% declined to participate, and ≈10% were either ineligible (e.g., because they used pesticides) or failed to fully enroll (Appendix Figure 1). By the end of the recruitment phase, an average of 34% (range 24%–44%) of the properties in a given neighborhood were enrolled in the project. Neither the proportion of properties enrolled (Appendix Table 3, Figure 1) nor the habitat composition of the neighborhoods (Appendix Tables 4, 5) varied significantly by treatment group.

When the study began, a mean of 101 (range 62–136) persons and 35 (range 14–58) outdoor pets were enrolled in each neighborhood, for a total of 2,384 human participants and 849 pets. Enrollment numbers did not vary significantly by treatment group (Table 1). On average, participants had a median age of 49 years, and 40% of households had an annual household income of $50,000–100,000 (Figure 1). Participants reported that when they spent time outside, most of their time was spent on their own properties or away from their neighborhoods (Appendix Figure 4). Participants reported regularly practicing just over 1 preventive behavior (e.g., tick checks) to protect themselves from ticks and TBDs (mean 1.2 + 0.3 SEM; Table 1).

**Table 1:** Characteristics of participants for the 24 residential neighborhoods together and for the 6 neighborhoods in each of the 4 treatment groups of tick-control interventions, New York, USA*

Characteristic	Overall	Neither active	Active Met52	Active bait boxes	Both active
No. neighborhoods	24	6	6	6	6
Mean no. human participants per neighborhood	97 (+ 19)	110 (+ 13)	94 (+ 26)	94 (+ 13)	90 (+ 18)
Mean no. outdoor pets per neighborhood	30 (+ 8)	26 (+ 9)	33 (+ 9)	29 (+ 5)	31 (+ 10)
Average median age of human participants, y	49 (+ 5)	48 (+ 4)	51 (+ 3)	48 (+ 6)	49 (+ 6)
Per capita no. preventive behaviors	1.27 (+ 0.27)	1.20 (+ 0.35)	1.37 (+ 0.27)	1.27 (+ 0.24)	1.27 (+ 0.24)
Self-reported cases of diagnosed TBDs per capita before study onset, 2011–2016	0.07 (+ 0.03)	0.05 (+ 0.02)	0.07 (+ 0.03)	0.07 (+ 0.02)	0.07 (+ 0.05)

*Data on age, previous cases of TBDs, and preventive behaviors were self-reported on the introductory survey administered during 2016–2017. Data on the number of participants and pets who spent time outside were averaged over the length of the study. Values in parentheses represent the standard error of the mean. TBDs, tickborne diseases.

### Tick Abundance

#### Questing Nymphal Ticks

**Figure 2:**
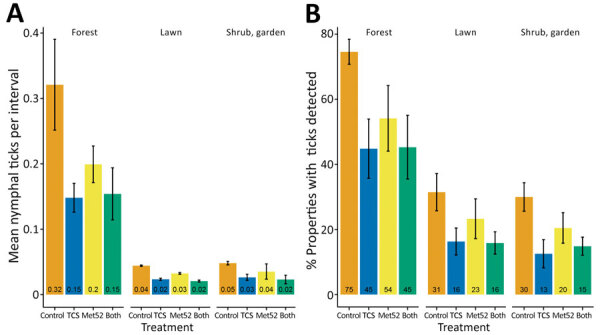
Detection of questing nymphal ticks during study of tick-control interventions in residential neighborhoods, New York, USA. A) Mean number of questing nymphal ticks per flagging interval (Appendix). B) Mean percentage of properties with questing nymphal ticks detected for each treatment group and in each habitat type (forest, lawn, shrub or garden). Totals are averaged over 3 years for each neighborhood. Data include ticks from the nymphal sampling period in May–July. Error bars represent SEM. TCS, Tick Control System.

Per sampling interval, more of the 4,040 questing nymphal ticks collected in the study were found in forested areas of properties than on lawns or in shrubs or gardens (Figure 2, panel A). At the neighborhood level of analysis, the presence of active TCS boxes was associated with a 53% reduction in the number of questing nymphal ticks in forest habitats compared with placebo controls, a statistically significant difference (Figure 2, panel A; Appendix Table 6). Despite an apparent reduction in tick abundance (compared with placebo controls) associated with Met52 treatment in forest habitats (Figure 2, panel A), this effect was not statistically significant, nor was there a significant effect of the 2 treatments used together (a significant interaction) (Appendix Table 6). Shrub and garden habitats showed a similar pattern; 40% fewer questing nymphal ticks were detected on properties with active TCS boxes than those with placebo controls (Figure 2, panel A; Appendix Table 7). This effect was statistically significant, but no significant effect of either active Met52 or the 2 treatments together was seen (Figure 2, panel A; Appendix Table 7). In lawn habitats at the neighborhood level of analysis, no statistically significant effect of either of the treatments used alone or together was seen (Figure 2, panel A; Appendix Table 8).

At the property level, ticks were detected in forested habitats on 75% of properties that received no active treatments but on only 45% of properties treated with active TCS boxes (Figure 2, panel B). A similar and statistically significant pattern was observed for the other 2 habitat types (Figure 2, panel B; Appendix Tables 9–11). There was no significant effect of active Met52 on the probability of detecting ticks in any of the 3 habitats, nor was there an effect of the treatments used together.

#### Larval and Nymphal Tick Burdens on Small Mammals

**Figure 3:**
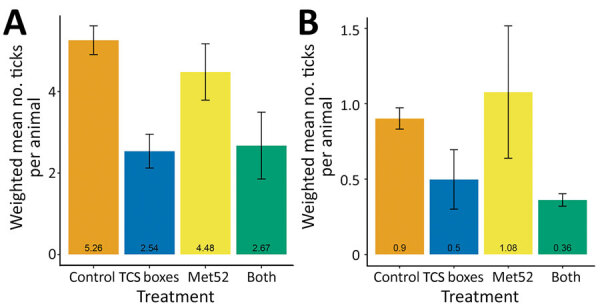
Weighted mean number of ticks on white-footed mice (A) and chipmunks (B) as a function of tick-control treatment, New York, USA, 2017–2019. Means represent the average of the 6 neighborhoods in each treatment group, whereas error bars represent SEs. Note that the scale of the y-axes differs. TCS, Tick Control System.

Averaged across all years and all treatments, white-footed mice had mean (+ SEM) tick burdens of 3.7 + 0.4 ticks/animal and chipmunks had 0.7 + 0.1 ticks/animal (Figure 3). The presence of active TCS boxes was associated with a reduction in the mean number of ticks per white-footed mouse by about half (Figure 3, panel A; Appendix Table 12). There was no significant effect of either active Met52 or the treatments together on the average tick burden on mice (Appendix Table 12). Neither treatment had a significant effect on the probability of tick presence on chipmunks or on nonzero tick burdens on chipmunks (Figure 3; Appendix Table 13).

### Case and Encounter Data for Humans

**Figure 4:**
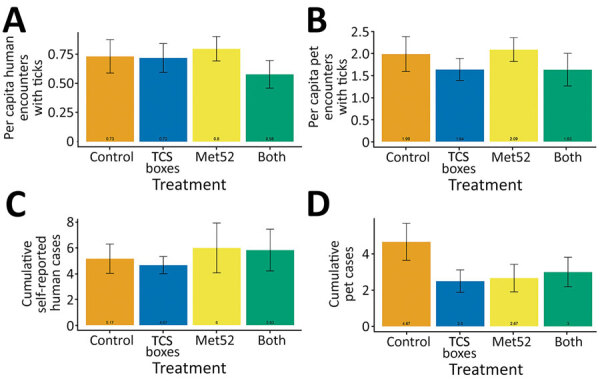
Mean per capita human and pet encounters with ticks and cumulative numbers of cases per neighborhood of tick-borne diseases for humans and pets in study of tick-control interventions, New York, USA. A) Human encounters; B) pet encounters; C) self-reported human cases; D) pet cases. Data represent the mean of the cumulative value (+ SEM) over the 4 years of treatments (2017–2020), averaged across neighborhoods in a treatment group. Note that the scale of the y-axes differs. TCS, Tick Control System.

We received 1,664 reports of encounters between ticks and human participants. The cumulative number of reported human encounters with ticks was ≈20% lower in neighborhoods treated with both active TCS boxes and active Met52, but this difference was not statistically significant (Figure 4, panel A), nor was there a significant effect of either of the active treatments alone (Appendix Table 14).

We received a total of 130 reports of TBD diagnoses in humans during 2017–2020. The active treatments, either alone or in combination, demonstrated no effect on the number of self-reported human cases of TBDs (Table 2; Figure 4, panel C; Appendix Table 15). We received permission to pursue confirmation for 84 (65%) of these cases and received 52 responses from healthcare providers. Of these, 35 (67%) confirmed diagnoses of a TBD. There was no significant effect of the active treatments, either alone or in combination, on the number of human cases of TBDs confirmed by healthcare providers (Table 2; Appendix Table 16).

**Table 2:** Cumulative diagnosed cases of tickborne diseases, averaged across the 6 residential neighborhoods in each treatment group of tick-control interventions, New York, USA*

Cases and treatment groups	Per capita cases (SE)	Cases/neighborhood (SE)	p value
Cases of diagnosed tickborne diseases in humans reported by participants, n = 130			
Control	0.05 (0.01)	5.17 (2.11)	
Active TCS boxes	0.05 (0.01)	4.67 (1.91)	NS
Active Met52	0.06 (0.02)	6.00 (2.45)	NS
Active TCS boxes and active Met52	0.06 (0.01)	5.83 (2.38)	NS
Cases of diagnosed tickborne diseases in humans confirmed by healthcare providers, n = 35†			
Control	0.009 (0.00)	1.00 (0.41)	
Active TCS boxes	0.012 (0.00)	1.17 (0.48)	NS
Active Met52	0.019 (0.01)	2.17 (0.88)	NS
Active TCS boxes and active Met52	0.016 (0.01)	1.50 (0.61)	NS
Cases of diagnosed tick-borne diseases in outdoor pets reported by participants, n = 77			
Control	0.17 (0.03)	4.67 (1.91)	
Active TCS boxes	0.08 (0.02)	2.50 (1.02)	‡
Active Met52	0.08 (0.03)	2.67 (1.09)	‡
Active TCS boxes and active Met52	0.11 (0.04)	3.00 (1.22)	NS

*For detailed statistical results, see Appendix Tables 16, 18, and 19 (https://wwwnc.cdc.gov/EID/article/28/5/21-1146-App1.pdf). Data represent the mean of the cumulative value (+SEM) over the 4 years of treatments, averaged across neighborhoods in a treatment group. NS, not significant. †Cases in humans confirmed by healthcare providers were less common than cases reported by participants because some participants did not grant permission to the investigators to pursue confirmation from healthcare providers, some healthcare providers did not respond to repeated requests for information, and some diagnoses from healthcare providers did not confirm patient reports. ‡Statistically significant differences.

### Case and Encounter Data for Pets

We received 1,307 reports of tick encounters for outdoor pets during 2017–2020. The cumulative number of reported pet encounters with ticks was ≈20% lower in neighborhoods with active TCS boxes, but this difference was not statistically significant, nor was there a significant effect of active Met52 treatments (Figure 4, panel B; Appendix Table 17). We received 77 reports of TBD diagnoses in pets during 2017–2020, as reported by owners. The incidence of owner-reported cases of TBDs in pets was lower by about half in neighborhoods with active TCS boxes or active Met52, and these differences were statistically significant (Table 2; Figure 4, panel D; Appendix Table 18).

### Effectiveness of Masking Procedures

Of 874 households participating in December 2020, a total of 507 primary contacts (58%) completed the final survey; 438 (86%) of those contacts said they did not know their neighborhood’s treatment assignment. Of the 65 who thought they knew their neighborhood’s treatment assignment, their guesses were incorrect (54%) more frequently than they were correct (46%) (Appendix).

## Discussion

We conducted a large-scale, randomized, masked, placebo-controlled study of the effects of 2 methods of tick control in residential neighborhoods. The central goal was to evaluate whether community-level control of ticks could reduce the threat of TBDs to public health. We documented significant reductions in tick abundance within certain treatment groups, most consistently within forest and garden habitats. These effects were not associated with significant reductions in human exposure to ticks or TBDs. However, TBD incidence in outdoor pets was significantly lower in neighborhoods that received the interventions.

Deploying of active TCS boxes in neighborhoods was associated with fewer questing nymphal ticks by >50% and fewer ticks on rodents by ≈50% compared with placebo controls. Active Met52 spray showed no effect on the abundance of either questing or attached ticks compared with placebo controls. Not surprisingly, using those 2 methods of tick control together did not show multiplicative effects, as indicated by the lack of statistically significant interactions between the interventions.

The protocols for TCS and Met52 used in this study complied with product labels. The low efficacy of Met52 may have arisen from degradation and low residual effects of the acaricide after applications (*29*). Other studies using TCS boxes or Met52 are not directly comparable to ours because they used multiple tick-control methods with unbalanced designs or lacked placebo controls (*20*,*21*,*30*), which are necessary to account for the presence of the food and shelter TCS boxes provide and to ensure that personnel collecting data are unaware of treatment assignments. Also, previous studies have tended to restrict TCS box placement or Met52 application to habitat edges, whereas we treated more broadly across habitat types. For example, a recent study placed TCS boxes in a single line along forest–lawn ecotones and found no effect (*31*). Keeping these important differences in mind, the reductions we observed in questing ticks and ticks on rodents in the neighborhoods with active TCS boxes and active Met52 were similar in magnitude to some previous studies using these tick-control methods (*22*) but differed from others (*15*,*20*,*31*,*32*).

Human encounters with ticks have been demonstrated to be a proxy for cases of TBD (*33*). We received 20% fewer cumulative reports of encounters between human participants and ticks, and between outdoor pets and ticks, in neighborhoods treated with both active TCS boxes and active Met52 than for placebo controls. However, this difference was not statistically significant, which might have been caused by stochastic variation among neighborhoods associated with relatively low numbers of cases.

The weak effects of tick reduction on tick encounters and reported cases of TBDs in humans could have arisen from one or more of the following reasons. First, despite persistent, energetic efforts throughout the first year of the study to recruit as many households as possible within neighborhoods, we enrolled 24%–44% of the households in each neighborhood (Appendix Figure 1). Although dozens of individual properties were treated per neighborhood, these treated areas might have been too sparse to provide added benefits over the treatment of individual properties. If more households in each neighborhood had participated, we might have observed greater reductions in tick numbers and an associated reduction in incidence of TBDs. However, increasing participation substantially in future interventions targeted at neighborhoods might not be feasible (Appendix Figure 1). General enthusiasm among residents was high, and the retention and response rates suggest high motivation among those who did participate.

Second, a total of 130 cases of TBDs were reported for all 24 neighborhoods cumulatively over the 4 years of treatments in this study, for a mean of only 5.5 cases per neighborhood. Such a low number of cases might have curtailed our ability to detect effects of the interventions. However, despite only 77 reported cases of TBDs in outdoor pets, or 3.7 cases per neighborhood on average, we detected a significant reduction in neighborhoods with active interventions compared with placebo controls. The absence of effects of treatment on incidence of self-reported and physician-confirmed cases of TBD in humans cannot be attributed solely to a limited number of cases.

A third possibility, related to the second, is that residents of our focal county frequently take actions to prevent exposure to tick bites and tickborne pathogens, which might have limited the effects observed from the interventions. Our study population within Dutchess County, New York, began experiencing high exposure to Lyme disease and other TBDs in the early 1990s, and many residents habitually engage in efforts to reduce risk, including use of repellents, protective clothing, tick checks, and yard management (*8*,*9*,*34*). In addition, awareness of relative risk might lead residents to spend more time in lawn and garden areas of their yards than in forested areas, where ticks were more abundant and the effects of treatments were stronger. These preventive behaviors could weaken the link between our tick-control interventions and disease incidence in the human population. If so, we would expect stronger effects of tick control in areas where residents demonstrate lower adherence to methods of personal protection. To examine this possibility, future studies could compare effectiveness of tick control interventions in areas of high and low adoption of personal protection measures.

The significant effect of active interventions observed for TBDs in outdoor pets but not in humans could have been caused by different patterns of space use (e.g., if outdoor pets spend more time in forested habitats within yards or use more of the neighborhood outside the individual property of residence). Use of repellents and other individual-based preventive measures might be less variable for pets than for humans, potentially increasing the ability to detect effects on pets. More information on how humans and pets use space, both within and outside residential areas, could help improve future tick-control interventions.

The observed effect of the active interventions on TBDs in outdoor pets should be interpreted cautiously. We observed no corresponding effect on tick encounters among pets, and we did not seek confirmation of pet diagnoses with veterinarians. Further, the incidence of TBDs in pets was the only outcome for which active Met52 treatments showed a significant effect.

In summary, although active TCS bait boxes were associated with reduced abundance of questing ticks, ticks attached to rodents, and TBD diagnoses in outdoor pets compared with placebo treatments, these interventions were not associated with significant reductions in human encounters with ticks or incidence of TBDs in humans. Thus, our study is consistent with that of Hinckley et al. (*23*) in suggesting that reducing the size of tick populations in residential areas might not result in strong effects on incidence of TBDs in human populations. More research is needed to address where in the environment, and under what conditions, humans most frequently encounter infected ticks, and in which geographic locations tick reductions will have the greatest impact on human health. One important conclusion for public health is that studies investigating tick reductions should also measure actual outcomes for people, such as disease incidence or tick encounters. 

Appendix Additional information about effects of tick-control interventions on tick abundance, human encounters with ticks, and incidence of tickborne diseases in residential neighborhoods, New York
