# Supplementary material for: Effects of Tick-Control Interventions on Tick Abundance, Human Encounters with Ticks, and Incidence of Tickborne Diseases in Residential Neighborhoods, New York, USA
Source: Emerg Infect Dis. 2022 May;28(5):957–66. doi: 10.3201/eid2805.211146 (PMC9045441; doi:10.3201/eid2805.211146)
Supplement: Appendix — Additional information about effects of tick-control interventions on tick abundance, human encounters with ticks, and incidence of tickborne diseases in residential neighborhoods, New York [file 21-1146-Techapp-s1.pdf]

Article DOI: https://doi.org/10.3201/eid2805.211146

Effects of Tick-Control Interventions on Tick Abundance, Human Encounters with Ticks, and Incidence of Tickborne Diseases in Residential Neighborhoods, New York

Appendix

Supplementary File: Detailed Methods

Study Design

We tested whether 2 tick-control interventions, used separately or together, affected tick abundance, tick encounters with humans and their pets, and cases of tickborne diseases in humans and pets. Interventions were imposed on all participating residential properties within a neighborhood, so that the unit of replication was the residential neighborhood (exceptions for several statistical analyses are described later). Each intervention had a placebo control, further described below. Study participants and scientific personnel that collected or managed data on response variables were masked to treatment assignment. The assignment of neighborhoods to the 4 treatment groups was randomized. Hence, our design was randomized, placebo-controlled, and masked.

Neighborhood Selection

We established 2 a priori criteria for selecting neighborhoods for inclusion in the study: (a) high incidence of Lyme disease (≈1% per year, which is 24× the overall incidence of Lyme disease for Dutchess County, New York, USA), and (b) moderate to high density of 1- and 2-family residences. The first criterion maximized the potential for detecting effects of interventions, and the second criterion increased feasibility of the research. We used georeferenced records of confirmed cases of Lyme disease reported during routine surveillance to the Dutchess County Department of Behavioral and Community Health (DCDBCH) during 2000–2011, totaling 8,100 cases. We selected 24 neighborhoods of ≈100 properties that contained a moderate to high density of 1- and 2-family homes and had high per capita Lyme

disease cases, while maintaining a minimum distance of 100 meters from other neighborhoods. Boundaries of neighborhoods were generally demarcated by physical features, such as large roads, commercial development, forested areas, or other nonresidential land-cover types.

Participant Recruitment

We contacted residents of target properties through numerous outreach methods, including phone calls, door-to-door personal visits, the placement of study information and consent materials at doors, and door hangtags. All adults (≥18 years of age) were considered eligible to be the primary contact for a household if they lived in free-standing homes within target neighborhoods with a surrounding yard and the authority to allow treatments to be deployed on that property. We excluded properties when residents did not reside primarily at the property to be treated or were not willing to forgo the use of their own acaricides or insecticides on their yards during the study period.

Property recruitment began in April 2016 and continued until June 2017, after which no new properties were enrolled in the study. When participants moved from a property during the study, the new occupants of the property were given the opportunity to enroll if the change of occupancy occurred before September 2020.

Introductory, Seasonal, and End-of-Study surveys

The primary contact for each participating household was asked to complete an introductory survey at the time of enrollment, during which they were asked to identify members of their households, as well as whether they had pets that spent time outside. The primary contact was also asked about prior diagnoses of tickborne diseases, outdoor activity levels and locations for all household members, adherence to preventive measures, and basic demographic information (including income, education, and age). We administered 4 additional seasonal surveys during the study to confirm that participants were still residing at properties. If participants responded affirmatively, we requested information about the number of persons and pets in residence at that time. At the conclusion of the study in December 2020, the primary contact was asked to complete a final survey, which included a question about whether they thought they knew the treatment group for their neighborhood.

Treatments

Beginning in spring 2017, we deployed 2 treatments, or their placebo equivalents, on participating properties: a spray containing spores of the F52 strain of the entomopathogenic fungus, *Metarhizium brunneum,* marketed as Met52® (Novozymes Biologicals, https://biosolutions.novozymes.com), and the Tick Control System (Select TCS, Tick Box Technology Corporation, http://www.tickboxtcs.com). TCS is a plastic box, which is placed inside a metal case that is staked to the ground. These boxes are designed to attract small mammals. Inside the box, the animals come into contact with a wick that applies the acaricide fipronil directly to their fur. Both interventions were selected on the basis of efficacy, as demonstrated in small-scale field trials (*1*–*5*), and commercial availability. The placebo control for Met52 was plain water, and the placebo control for TCS boxes were identical boxes that contained no fipronil.

For Met52 preparation for neighborhoods receiving active Met52 treatments, truck-mounted high-pressure sprayers (GNC Industries, Inc., https://gncindustries.com) were filled with 1,893 L (500 gallons) of water, into which Met52 spore suspension was added at a concentration of 2.22 L/378.5 L (100 gallons) of water and mixed thoroughly for a final concentration consistent with product label instructions. Identical trucks and sprayers were filled with water to treat the properties receiving placebo controls. All sprayers were filled at locations distant from the neighborhoods to be treated. Met52 or plain water was sprayed on all participating properties within designated neighborhoods at a pressure of 1.2– 1.4 mega Pascals (175–200 pounds per square inch), and at a rate of 4 L of spray per 93 m^2 (1,000 square feet). The sprayer apparatus, including the tank, was rinsed 3× with water after every neighborhood treatment. All vegetation on the property from ground level to 1 meter above the ground was sprayed according to product directions (e.g., avoiding water features). We also allowed residents to specify areas (e.g., vegetable gardens) not to be sprayed (n = 82 exclusion areas on 1,002 properties). In addition, when properties contained extensive forested areas, spraying extended 12 meters into the forest from the lawn or shrub and garden areas bordering the forest. Spraying was conducted twice each year to correspond to the periods immediately before and during the peak of questing activity of nymph-stage blacklegged ticks (*6*) (Appendix Table 1). We randomized the order in which each treatment group was sprayed (Appendix Table 1). Spraying was suspended during rain events and resumed within 24 hours thereafter.

The average property was treated with 5.9 (range 3–26) TCS boxes, or the equivalent number of placebo controls, which is a density of ≈38 boxes/hectare. Boxes were placed at least 10 meters apart in all habitat types that we sampled for ticks and were deployed in protected locations, such as along building foundations and under vegetation, that are frequently used by small mammals. Boxes were covered with galvanized steel shrouds and anchored in place with nylon zip ties attached to 31-cm long galvanized ground stakes (McGregor Fence Company, https://www.mcgregorfence1.com) to prevent disturbance by larger mammals. TCS boxes were deployed in 2 rounds each year, corresponding to the activity peaks for nymphal and larval blacklegged ticks (*6*), which ensured that the fipronil acaricide and bait were not depleted (Appendix Table 1).

Habitat Characterization

To determine habitat characteristics of the neighborhoods, we used publicly available statewide digital orthoimagery of Dutchess County (*7*) taken in the spring of 2016. We classified every pixel in each neighborhood by using the maximum-likelihood classification tool in ArcMap software version 10.4 (https://www.esri.com). To estimate forest habitat, we used a Geographic Information System (GIS) layer developed in 2017 by Robert S. Wills, Senior GIS Project Coordinator with Dutchess County Department of Planning and Development. We estimated error rates for all pixel classifications by selecting a random subset of 10% of the properties in each neighborhood and manually reviewing the output of the classification of the pixels on that property. The mean error rate for classification of lawn habitat by the maximum-likelihood classification tool was 12.9%. We considered this too high and opted instead to combine lawn/shrub/garden/field into 1 classification.

Tick Abundance: Questing Ticks

Each year, beginning in the late spring of 2017 through 2019, we sampled for ticks at 20 randomly selected properties within each neighborhood during the nymphal peak in blacklegged tick activity (*6*) in May–July. Sampling was not completed in 2020 because of the coronavirus disease pandemic. We sampled in 3 habitat types on each property: lawn, forest, and shrub or garden, whenever these habitat types were present. Because of variation in the size and arrangement of habitat features on properties, we performed timed flagging of each of the habitat types. We used 1-meter by 1-meter white corduroy cloth to flag-sample ticks modified from Rulison et al. (*8*), conducting up to ten 30-second intervals of flagging in each habitat type. We

avoided resampling and inspected the flagging cloth after each 30-second interval to count and collect all ticks. Researchers also counted and collected all ticks detected in self-inspections of field attire (white coveralls) at the end of each interval. Flagging was conducted between 9 AM and 5:30 PM each sampling day. We did not sample when it was raining or when vegetation was wet.

Tick Abundance: Tick Burdens on Small Mammals

To assess tick burdens on small mammals, we conducted mark-recapture sampling by using Sherman live traps (7.6 × 8.9 × 22.9 cm) at 10 randomly selected properties in each of the 24 neighborhoods from August through mid-to-late September annually during 2017–2019, corresponding to the activity peak of the larval stage (*6*). We set 18 traps on each property, with 2 traps at each of 9 trap stations and trap stations at least 5 meters apart, and checked them for 3 consecutive mornings. We baited traps with a 3:1 mixture of crimped oats and black oil sunflower seeds. We focused our trapping efforts and data collection on the white-footed mouse (*Peromyscus leucopus*) and the eastern chipmunk (*Tamias striatus*) because of their importance in transmitting the Lyme bacterium to ticks (*9*). We recorded the number of larval and nymphal ticks that we observed on an individual animal's head and ears, which prior research has demonstrated to accurately represent the total body burden (*10*). Each individual mouse or chipmunk was given a monel ear tag (style 1005–1, National Band & Tag Company, https://www.nationalband.com) with a unique identification number. Ticks were counted only upon the first capture of each individual animal. We released all animals at the point of capture, including nontarget species, which we released without handling.

Case and Encounter Data for Humans and Pets

To collect data on cases of tickborne diseases experienced by participating humans and their pets, and on human and pet encounters with ticks, we distributed biweekly surveys to each participating household. Every 2 weeks (Appendix Table 2), we contacted the primary contact person for each participating household to ask whether they or any other full-time resident of their household, including pets that spent time outdoors, had encountered a tick or had a diagnosis of a tickborne disease in the previous 2 weeks. If a participant answered yes to the initial question, they were prompted to complete a follow-up survey.

Follow-up surveys requested further information on human or pet encounters with ticks. Participants reporting tickborne illness in a person were asked to consider signing a medical consent form and completing a Health Insurance Portability and Accountability Act (HIPAA) release enabling us to request confirmation of the case by their healthcare provider. If we received a completed medical consent and HIPAA release, designated staff members contacted the healthcare provider's office to request an abstraction of the diagnosis.

Informed Consent

The Institutional Review Board of the Cary Institute of Ecosystem Studies in Millbrook, NY, USA reviewed and approved protocols (approval no. 131–2016) involving human subjects. All participants were fully informed of study procedures, their legal rights and responsibilities, the general scientific benefits expected from the research, and their right for voluntary termination without penalty or censure.

Animal Care and Handling

Protocols for the live-trapping and handling of small mammals were approved by the Cary Institute Institutional Animal Care and Use Committee (IACUC Code 02–16). Trapping was conducted under License to Collect or Possess: Scientific #1512 from the New York State Department of Environmental Conservation.

Data Analysis

Data on participants were stored in Salesforce (https://www.salesforce.com), with password-protected access that masked confidential and sensitive information. Access to data was provided to personnel only as needed, and all persons who collected, entered, or worked with raw data were unaware of treatment assignments. Field data on ticks and rodents were recorded in Microsoft Excel (https://www.microsoft.com). Data were analyzed in R version 4.0.1 (*11*), using the packages *tidyr* (*12*), *dplyr* (*13*), and *forcats* (*14*) to format and manipulate data, and the packages *ggplot2* (*15*) and *cowplot* (*16*) for graphing. We used the *broom* (*17*) and *broom.mixed* (*18*) packages to tidy statistical output. We analyzed data on the proportion of each neighborhood that comprised forest and nonforest (i.e., shrub/lawn/garden) habitat by using linear models, with treatment as a factor, checking that assumptions of tests were met.

Tick Abundance: Questing Ticks

We analyzed data on the abundance of questing nymphal ticks in May–early July over a 3-year period beginning in 2017, the year treatments were first applied to the properties. Tick densities were estimated as the mean number of questing nymphal ticks per flagging interval, with each habitat type (forest, lawn, shrub/garden) evaluated separately. We sampled ticks twice on each property during each year's nymphal activity period, and we estimated tick density on a property as the maximum of the 2 samples. For 12 of 10,778 occasions, the number of intervals in a particular habitat type on a particular property was not recorded. In these cases, for the particular habitat type, we used the number of intervals recorded during the second visit of the year to the same property.

We evaluated our data on questing ticks in 2 ways. First, we analyzed data at the neighborhood level, using the mean of the maximum number of questing nymphal ticks collected per interval from each sampled property within a neighborhood, averaged for each neighborhood. Because we sampled the same neighborhoods over 3 years, we treated neighborhood as a random effect, with year, treatment, and an interaction between the presence of active TCS boxes and active Met52 treatments as fixed effects. We used linear mixed-effects models built by using package *nlme* (*19*) to conduct these analyses, transforming the data when needed to conform to assumptions of tests.

In our second analysis, we analyzed data from each of the individual properties we sampled, without averaging these data for the entire neighborhood. For these property-level analyses, we only included data from properties at which we were able to conduct fieldwork in all 3 years: 2017, 2018, and 2019. Each neighborhood had a minimum of 10 properties with 3 complete years of sampling (mean 15.8 $\pm$ 0.6 SEM). For flagging data, zero values were common, representing 43%–80% of our property-level data, depending on habitat type. To evaluate these data at the property level, we used generalized linear mixed models with a binomial distribution and a logit link function to fit a logistic model to the zero values versus the nonzero values. These analyses were conducted with the *glmmTMB* function in package *glmmTMB* (*20*). We tested whether our data met the assumptions of our models by using the *DHARMa* package (*21*). We did not attempt to fit a generalized linear model to the nonzero values, as in a hurdle modeling approach, because the nonzero values did not conform to the assumptions of distributions (e.g., gamma, gaussian).

Tick Abundance: Tick Burdens on Small Mammals

For data on the tick burdens on white-footed mice (*Peromyscus leucopus*) and eastern chipmunks (*Tamias striatus*), we calculated the weighted mean of the number of larval and nymphal ticks on each species on each property by using the number of unique individual animals (mice or chipmunks) on each property as the weighting factor. More than 98% of ticks were larvae, with <2% nymphs. Means of tick burdens did not include any small mammals that escaped during handling or any small mammals that had been previously trapped at a different property within that season. Some properties in a neighborhood had no captures of mice or no captures of chipmunks in a given year. For these properties, there was no estimate of ticks on that species of rodent, and these properties were excluded from calculations of that neighborhood's mean tick burdens for that year.

Because we sampled the same neighborhoods over 3 years, we treated neighborhood as a random effect in our statistical models, with year, treatment, and an interaction between the presence of active TCS boxes and active Met52 treatments as fixed effects. The weighted means of ticks on mice were analyzed by using linear mixed effects models with package *nlme* (*19*), using a square-root transformation of the data to conform to assumptions of tests. The weighted means of ticks on chipmunks did not conform to the assumptions of linear models, so we first used a generalized linear mixed model with a binomial distribution and a logit link function to fit a logistic model to the zero values versus the nonzero values. We then used a generalized linear mixed model with a gamma distribution for the nonzero values, with neighborhood as a random effect, and year, treatment, and an interaction between the use of active TCS boxes and/or Met52 spray as fixed effects. These analyses were conducted with the *glmmTMB* function in package *glmmTMB* (*20*). We tested whether our data met the assumptions of the models by using the *DHARMa* package (*21*).

Case and Encounter Data for Humans and Pets

Our data for encounters with ticks and cases of tickborne diseases for both humans and pets were gathered from a total of 78 biweekly surveys, which we administered to participants during May 2017–December 2020 (Appendix Table 2). For all case and encounter data, we included in our analyses estimates of the number of participating persons or pets in a neighborhood over a particular year. The number of persons and pets was determined by surveys conducted at the start or end of each field season. If a participating household did not respond to

a specific survey, we assumed the number of persons and pets remained the same as in the previous survey. We used these data to calculate the number of human and pet participants in a neighborhood during each biweekly survey period and then averaged these biweekly estimates to establish the annual number of participants. If participants informed us of changes to the number of residents or pets in their household during a season, these changes were incorporated at the time of the relevant biweekly survey. Members of households that withdrew from the study were not included in analyses of cases for seasons in which they were enrolled for fewer than half of the biweekly surveys.

We calculated the cumulative number of reported encounters by humans or pets for all participating households in a neighborhood on the basis of participant responses to our biweekly surveys. A specific tick encounter reported by a participant was calculated as a binary value (yes/no), regardless of the number of ticks the participant reported having encountered. To be included in a particular year's data on encounters or cases, a household needed to have responded to ≥1 of that year's biweekly surveys.

We analyzed the number of human cases in 2 ways. First, we considered self-reported cases of tickborne diseases indicated by participants during biweekly surveys. For these self-reports, participants were asked to report household cases of tickborne diseases diagnosed by a healthcare provider. When participants granted us permission to contact their healthcare provider, we did so, using the clinical diagnosis of a case as indicated by the healthcare provider as the basis for a confirmed case. For cases in pets, we relied on reports of diagnosed cases from owners through the biweekly surveys.

For reported tick encounters and cases of tickborne diseases for humans and pets, we calculated the cumulative number of reports of each type for each neighborhood for the 4 years of treatments. Because our data relied on responses to our biweekly surveys, our estimates of the number of participants in a neighborhood included only participants or pets from households that submitted ≥1 biweekly survey in a given year. We used generalized linear models with treatment and an interaction between the presence of active TCS boxes and active Met52 as fixed effects, with an offset for the mean number of humans (or pets) in a neighborhood. We used the *glmmTMB* function in package *glmmTMB* (*20*) to fit models with a negative binomial distribution. We evaluated the residuals of the models (e.g., for overdispersion) by using the

DHARMa package (*21*). When necessary to account for a large number of zero values in a particular response variable, we included a term for zero-inflation.

Supplementary Results

Study Participants

When the study began, a mean of 101 persons (range 62–136) and 35 outdoor pets (range 14–58) were enrolled in each neighborhood, for a total of 2,384 human participants and 849 pets. Because of attrition, an average of 7% fewer households were enrolled by the end of the study, reaching a mean of 95 participants per neighborhood (range 61–124) (Appendix Figure 2). Approximately two thirds of the primary contacts for each household reported educational attainment of a college degree or higher (Appendix Figure 3).

Approximately 80% of participating households responded to the biweekly survey regarding tick encounters and disease diagnoses, and 83% responded to at least half of the surveys each season. Ninety-six percent responded to ≥1 of the biweekly surveys in any given season.

Effectiveness of Masking Procedures

Of 874 households participating in December 2020, 507 primary contacts (58%) completed the final survey and 438 of these (86%) said they did not know their neighborhood's treatment assignment. Of the 65 that thought they knew their neighborhood's treatment assignment, their guesses were incorrect (54%) more frequently than they were correct (46%). Of the 13% (N = 65) who said they thought they knew ≥1 of the treatment assignments, most thought they knew either the type of Met52 treatment they received (43%) or the types of both treatments (40%). Only 17% thought they knew only the type of bait boxes their neighborhood was assigned. Of those who thought they knew the type of Met52 treatment they received, 15 of 28 (53%) were correct, and there was no statistically significant association between their guess and their actual treatment category based on a Fisher exact test ($\chi^2 = 0.53$; $p>0.05$).

References

1. Schulze TL, Jordan RA, Schulze CJ, Healy SP. Suppression of *Ixodes scapularis* (Acari: Ixodidae) following annual habitat-targeted acaricide applications against fall populations of adults. J Am Mosq Control Assoc. 2008;24:566–70. PubMed https://doi.org/10.2987/08-5761.1

2. Williams SC, Little EAH, Stafford KC III, Molaei G, Linske MA. Integrated control of juvenile *Ixodes scapularis* parasitizing *Peromyscus leucopus* in residential settings in Connecticut, United States. Ticks Tick Borne Dis. 2018;9:1310–6. PubMed https://doi.org/10.1016/j.ttbdis.2018.05.014

3. Williams SC, Stafford KC III, Molaei G, Linske MA. Integrated control of nymphal *Ixodes scapularis*: Effectiveness of white-tailed deer reduction, the entomopathogenic fungus *Metarhizium anisopliae*, and fipronil-based rodent bait boxes. Vector Borne Zoonotic Dis. 2018;18:55–64. PubMed https://doi.org/10.1089/vbz.2017.2146

4. Little EAH, Williams SC, Stafford KC III, Linske MA, Molaei G. Evaluating the effectiveness of an integrated tick management approach on multiple pathogen infection in *Ixodes scapularis* questing nymphs and larvae parasitizing white-footed mice. Exp Appl Acarol. 2020;80:127–36. PubMed https://doi.org/10.1007/s10493-019-00452-7

5. Schulze TL, Jordan RA, Williams M, Dolan MC. Evaluation of the SELECT tick control system (TCS), a host-targeted bait box, to reduce exposure to *Ixodes scapularis* (Acari: Ixodidae) in a Lyme disease endemic area of New Jersey. J Med Entomol. 2017;54:1019–24. PubMed https://doi.org/10.1093/jme/tjx044

6. Levi T, Keesing F, Oggenfuss K, Ostfeld RS. Accelerated phenology of blacklegged ticks under climate warming. Philos Trans R Soc Lond B Biol Sci. 2015;370:370. PubMed https://doi.org/10.1098/rstb.2013.0556

7. New York State Digital Orthoimagery Program. Dutchess County direct download (2016). 2016 [cited 2021 Mar 18]. http://gis.ny.gov/gateway/mg/2016/dutchess

8. Rulison EL, Kuczaj I, Pang G, Hickling GJ, Tsao JI, Ginsberg HS. Flagging versus dragging as sampling methods for nymphal *Ixodes scapularis* (Acari: Ixodidae). J Vector Ecol. 2013;38:163–7. PubMed https://doi.org/10.1111/j.1948-7134.2013.12022.x

9. LoGiudice K, Ostfeld R, Schmidt K, Keesing F. The ecology of infectious disease: effects of host diversity and community composition on Lyme disease risk. Proc Natl Acad Sci USA. 2003;100:567–71. **PMID 12525705**

10. Schmidt KA, Ostfeld RS, Schauber EM. Infestation of *Peromyscus leucopus* and *Tamias striatus* by *Ixodes scapularis* (Acari: Ixodidae) in relation to the abundance of hosts and parasites. J Med Entomol. 1999;36:749–57. PubMed https://doi.org/10.1093/jmedent/36.6.749

11. R Core Team. R: A language and environment for statistical computing. Vienna, Austria: R Foundation for Statistical Computing; 2020 [cited 2021 Mar 18]. https://www.r-project.org/

12. Wickham H, Henry L. tidyr: Tidy messy data. R package version 1.1.0. 2020 [cited 2021 Mar 18]. https://cran.r-project.org/web/packages/tidyr/index.html

13. Wickham H, Francois R, Henry L, Muller K. dplyr: A grammar of data manipulation. R package version 1.0.0. 2020 [cited 2021 Mar 18]. https://cran.r-project.org/package=dplyr

14. Wickham H. forcats: Tools for working with categorical variables (factors). R package version 0.5.0. 2020 [cited 2021 Mar 18]. https://cran.r-project.org/package=forcats

15. Wickham H. ggplot2: Elegant graphics for data analysis. New York, NY: Springer-Verlag; 2016 [cited 2021 Mar 18]. https://ggplot2.tidyverse.org

16. Wilke CO. cowplot: Streamlined plot theme and plot annotations for “ggplot2”. 2017 [cited 2021 Mar 18]. https://cran.r-project.org/package=cowplot

17. Robinson D, Hayes A. broom: Convert statistical analysis objects into tidy tibbles. R package version 0.5.6. 2020 [cited 2021 Mar 18]. https://cran.r-project.org/package=broom

18. Bolker B, Robinson D. broom.mixed: Tidying Methods for Mixed Models. R package version 0.2.6. 2020 [cited 2021 Mar 18]. https://cran.r-project.org/package=broom.mixed

19. Pinheiro J, Bates D. DebRoy S, Sarkar D, R Core Team. nlme: Linear and nonlinear mixed effects models. R package version 3.1–148. 2020 [cited 2021 Mar 18]. https://cran.r-project.org/package=nlme

20. Brooks ME, Kristensen K, van Benthem KJ, Magnusson A, Berg CW, Nielsen A, et al. glmmTMB balances speed and flexibility among packages for zero-inflated generalized linear mixed modeling. R J. 2017;9:378–400. https://doi.org/10.32614/RJ-2017-066

21. Hartig F. DHARMa: residual diagnostics for hierarchical (multi-level/mixed) regression models. R package version 0.3.3.0. 2020. https://cran.r-project.org/package=DHARMa

Appendix Table 1. Dates of tick-control treatments for both TCS boxes and Met52 spray, New York, 2017–2020*

Year	Order	TCS box and Met52 deployment	Met52	TCS box pick up and deployment	TCS box pick up
2017	*a-b-c-d*	12 April–5 May	1–28 June	5 July–4 Aug	19 Oct–17 Nov
2018	*d-c-b-a*	12 April–8 May	1–27 June	5 July–1 Aug	5–31 Oct
2019	*c-d-a-b*	12 April–13 May	3–28 June	5–31 July	7–31 Oct
2020	*b-d-a-c*	13 April–18 May	1–30 June	6–31 July	5–29 Oct

*To avoid bias in the sequence of visitation for each treatment category, neighborhoods in the 4 treatment groups (± active TCS boxes and ± active Met52 spray) were visited in a different order each year, as indicated in the second column. TCS, Tick Control System.

Appendix Table 2. Annual number of biweekly surveys in study of tick-control interventions in residential neighborhoods, New York, with start and end dates, 2017–2020

Year	No. biweekly surveys	First biweekly survey	Last biweekly survey
2017	15	2 May	14 November
2018	19	27 March	4 December
2019	22	19 February	10 December
2020	22	18 February	8 December

Appendix Table 3. Results of linear model of the proportion of households in each neighborhood that were enrolled in study of tick-control interventions in residential neighborhoods, New York, 2017, with treatment as a factor*

Term	Estimate	SE	t statistic	p value
(Intercept)	0.35	0.02	15.02	0.00
Active Met52	0.00	0.03	−0.01	0.99
Active TCS boxes	−0.01	0.03	−0.41	0.69
Active TCS boxes and active Met52	−0.02	0.03	−0.63	0.53

*TCS, Tick Control System.

Appendix Table 4. Results of linear model of proportion of each neighborhood comprised of forest in study of tick-control interventions in residential neighborhoods, New York, with treatment as a factor*

Term	Estimate	SE	t statistic	p value
(Intercept)	0.46	0.05	9.06	<<0.01
Active Met52	−0.02	0.07	−0.21	0.84
Active TCS boxes	0.012	0.07	0.16	0.87
Active TCS boxes and active Met52	−0.09	0.07	−1.19	0.25

*TCS, Tick Control System.

Appendix Table 5. Results of linear model of proportion of each neighborhood comprised of non-forest habitat (lawn, shrub, garden) in study of tick-control interventions in residential neighborhoods, New York, with treatment as a factor*

Term	Estimate	SE	t statistic	p value
(Intercept)	0.29	0.03	8.30	0.00
Active Met52	0.01	0.05	0.24	0.81
Active TCS boxes	−0.02	0.05	−0.41	0.69
Active TCS boxes x Met52	0.06	0.05	1.13	0.27

*TCS, Tick Control System.

Appendix Table 6. Results of linear mixed-effects model of the average maximum number of questing nymphal ticks per interval in forested habitat, New York, 2017–2019, analyzed at the neighborhood level, with year, treatments, and an interaction between active TCS boxes and active Met52 as fixed effects and neighborhood as a random factor*

Effect	Group	Term	Estimate	SE	df	t value	p value
Fixed	Fixed	(Intercept)	82.05	29.16	47.00	2.81	0.01
Fixed	Fixed	Year	−0.04	0.01	47.00	−2.80	0.01*
Fixed	Fixed	Active TCS boxes	−0.20	0.09	20.00	−2.24	0.04*
Fixed	Fixed	Active Met52	−0.15	0.09	20.00	−1.64	0.12
Fixed	Fixed	Active TCS boxes and active Met52	0.17	0.13	20.00	1.36	0.19
Random	Neighborhood	sd (Intercept)	0.14				
Random	Residual	sd Observation	0.10				

*TCS, Tick Control System.

Appendix Table 7. Results of linear mixed-effects model of the average maximum number of questing nymphal ticks per interval in shrub and garden habitat, New York, 2017–2019, analyzed at the neighborhood level, with year, treatments, and an interaction between active TCS boxes and active Met52 as fixed effects and neighborhood as a random factor*

Effect	Group	Term	Estimate	SE	df	t value	p value
Fixed	fixed	(Intercept)	−43.66	19.19	47.00	−2.27	0.03
Fixed	fixed	Year	0.02	0.01	47.00	2.29	0.03*
Fixed	fixed	Active TCS boxes	−0.08	0.04	20.00	−2.15	0.04*
Fixed	fixed	Active Met52	−0.05	0.04	20.00	−1.34	0.20
Fixed	fixed	Active TCS boxes x Met52	0.06	0.05	20.00	1.09	0.29
Random	Neighborhood	sd (Intercept)	0.05				
Random	Residual	sd Observation	0.07				

*TCS, Tick Control System.

Appendix Table 8. Results of linear mixed-effects model of the average maximum number of questing nymphal ticks per interval in lawn habitat, New York, 2017–2019, analyzed at the neighborhood level, with year, treatments, and an interaction between active TCS boxes and active Met52 as fixed effects and neighborhood as a random factor*

Effect	Group	Term	Estimate	SE	df	t value	p value
Fixed	fixed	(Intercept)	−13.79	15.48	47.00	−0.89	0.38
Fixed	fixed	Year	0.01	0.01	47.00	0.90	0.37
Fixed	fixed	Active TCS boxes	−0.06	0.04	20.00	−1.70	0.10
Fixed	fixed	Active Met52	−0.04	0.04	20.00	−1.06	0.30
Fixed	fixed	Active TCS boxes x Met52	0.03	0.05	20.00	0.55	0.59
Random	Neighborhood	sd (Intercept)	0.05				
Random	Residual	sd Observation	0.05				

*TCS, Tick Control System.

Appendix Table 9. Results of generalized linear mixed-effects model of the detection of questing nymphal ticks in forest habitat, New York, 2017–2019, analyzed at the property level, with year, treatments, and an interaction between active TCS boxes and active Met52 as fixed effects, and property within neighborhood as a random effect*

Effect	Group	Term	Estimate	SE	z value	p value
Fixed		(Intercept)	1.59	0.49	3.24	0.00
Fixed		Year	−0.08	0.09	−0.82	0.41
Fixed		Active TCS boxes	−1.68	0.64	−2.64	0.01*
Fixed		Active Met52	−1.14	0.64	−1.78	0.07
Fixed		Active TCS boxes x Met52	1.10	0.90	1.22	0.22
Random	Property	sd (Intercept)	1.18			
Random	Neighborhood	sd (Intercept)	0.99			

*TCS, Tick Control System.

Appendix Table 10. Results of generalized linear mixed-effects model of the detection of questing nymphal ticks in shrub and garden habitat, New York, 2017–2019, analyzed at the property level, with year, treatments, and an interaction between active TCS boxes and active Met52 as fixed effects, and property within neighborhood as a random effect*

Effect	Group	Term	Estimate	SE	z value	p value
Fixed		(Intercept)	−1.60	0.33	−4.91	0.00
Fixed		Year	0.30	0.09	3.20	0.00*
Fixed		Active TCS boxes	−1.30	0.38	−3.37	0.00*
Fixed		Active Met52	−0.59	0.37	−1.61	0.11
Fixed		Active TCS boxes x Met52	0.85	0.54	1.56	0.12
Random	Property	sd (Intercept)	0.73			
Random	Neighborhood	sd (Intercept)	0.50			

*TCS, Tick Control System.

Appendix Table 11. Results of generalized linear mixed-effects model of the detection of questing nymphal ticks in lawn habitat, New York, 2017–2019, analyzed at the property level, with year, treatments, and an interaction between active TCS boxes and active Met52 as fixed effects, and property within neighborhood as a random effect*

Effect	Group	Term	Estimate	SE	z value	p value
Fixed		(Intercept)	−1.08	0.36	−3.00	0.00
Fixed		Year	0.06	0.09	0.71	0.48
Fixed		Active TCS boxes	−1.00	0.45	−2.22	0.03*
Fixed		Active Met52	−0.51	0.44	−1.15	0.25
Fixed		Active TCS boxes x Met52	0.48	0.64	0.75	0.45
Random	Property	sd (Intercept)	0.77			
Random	Neighborhood	sd (Intercept)	0.66			

*TCS, Tick Control System.

Appendix Table 12. Results of linear mixed-effects model of the weighted mean number of ticks on white-footed mice, New York,with year, treatment, and an interaction between active TCS boxes and active Met52 as fixed effects and neighborhood as a random effect*

Effect	Group	Term	Estimate	SE	df	t value	p value
Fixed	fixed	(Intercept)	−340.05	222.00	47	−1.53	0.13
Fixed	fixed	Year	0.17	0.11	47	1.54	0.13
Fixed	fixed	Active TCS boxes	−0.80	0.34	20	−2.37	0.03*
Fixed	fixed	Active Met52	−0.26	0.34	20	−0.77	0.45
Fixed	fixed	Active TCS boxes x Met52	0.26	0.48	20	0.55	0.59
Random	Neighborhood	sd_(Intercept)	0.39				
Random	Residual	sd_Observation	0.76				

*TCS, Tick Control System.

Appendix Table 13. Results of hurdle modeling of the weighted mean of the number of ticks on chipmunks, New York*

Zero versus nonzero values of the weighted mean of ticks on chipmunks†						
Fixed		(Intercept)	23.06	21235.89	0.00	1.00
Fixed		2018	2.77	1.48	1.87	0.06
Fixed		2019	2.77	1.48	1.87	0.06
Fixed		Active TCS boxes	−23.46	21235.89	0.00	1.00
Fixed		Active Met52	−19.92	21235.89	0.00	1.00
Fixed		Active TCS boxes x Met52	19.22	21235.89	0.00	1.00
Random	Neighborhood	sd__(Intercept)	2.19			
Positive values of the weighted mean of ticks on chipmunks‡						
Fixed		(Intercept)	−0.23	0.33	−0.70	0.49
Fixed		2018	−0.28	0.29	−0.97	0.33
Fixed		2019	0.25	0.30	0.83	0.41
Fixed		Active TCS boxes	−0.12	0.44	−0.27	0.79
Fixed		Active Met52	0.01	0.41	0.02	0.98
Fixed		Active TCS boxes x Met52	−0.18	0.63	−0.28	0.78
Random	Neighborhood	(Intercept)	0.50			

*TCS, Tick Control System.
†Results of generalized linear model of the zero versus nonzero values for the weighted means, fit to a logistic distribution with year and an interaction between active TCS boxes and active Met52 as fixed effects and neighborhood as a random effect.
‡Results of generalized linear model of the nonzero values for the weighted means, fit with a gamma distribution, with year and an interaction between active TCS boxes and active Met52 as fixed effects and neighborhood as a random effect.

Appendix Table 14. Results of generalized linear model of cumulative self-reported encounters with ticks by human participants in tick-control intervention study, New York, 2017–2020, fit with a negative binomial distribution and an offset for the number of human participants in a neighborhood*

Term	Estimate	SE	z value	p value
(Intercept)	−0.34	0.15	−2.24	0.03
Active Met52	0.01	0.22	0.05	0.96
Active TCS boxes	0.15	0.21	0.73	0.47
Active TCS boxes x Met52	−0.38	0.32	−1.17	0.24

*TCS, Tick Control System.

Appendix Table 15. Results of generalized linear model of cumulative self-reported cases of tickborne diseases in humans, New York, 2017–2020, fit with a negative binomial distribution and an offset for the number of human participants in a neighborhood*

Term	Estimate	SE	z value	p value
(Intercept)	−3.07	0.21	−14.53	0.00
Active Met52	0.09	0.30	0.30	0.76
Active TCS boxes	0.30	0.29	1.02	0.31
Active TCS boxes x Met52	−0.05	0.41	−0.11	0.91

*TCS, Tick Control System.

Appendix Table 16. Results of generalized linear model of cumulative cases of tickborne diseases in humans, New York, 2017–2020, as confirmed by a healthcare provider*

Term	Estimate	SE	z value	p value
(Intercept)	−4.68	0.45	−10.31	0.00
Active Met52	0.36	0.60	0.59	0.55
Active TCS boxes	0.82	0.57	1.43	0.15
Active TCS boxes x Met52	−0.59	0.79	−0.75	0.46

*The model is fit with a negative binomial distribution and an offset for the number of participating persons in a neighborhood. TCS, Tick Control System.

Appendix Table 17. Results of generalized linear model of cumulative pet encounters with ticks, New York, 2017–2020, fit with a negative binomial distribution and an offset for the number of participating pets in a neighborhood*

Term	Estimate	SE	z value	p value
(Intercept)	0.59	0.16	3.79	0.00
Active Met52	−0.08	0.22	−0.35	0.73
Active TCS boxes	0.14	0.20	0.69	0.49
Active TCS boxes x Met52	−0.29	0.30	−0.96	0.34

*TCS, Tick Control System.

Appendix Table 18. Results of generalized linear model of cumulative diagnosed cases of tickborne diseases in pets, New York, 2017–2020, fit with a Poisson distribution and with an offset for the number of participating pets in a neighborhood*

Term	Estimate	SE	z value	p value
(Intercept)	−1.81	0.19	−9.56	0.00
Active Met52	−0.67	0.32	−2.11	0.04*
Active TCS boxes	−0.70	0.31	−2.25	0.02*
Active TCS boxes x Met52	0.76	0.47	1.61	0.11

*TCS, Tick Control System.

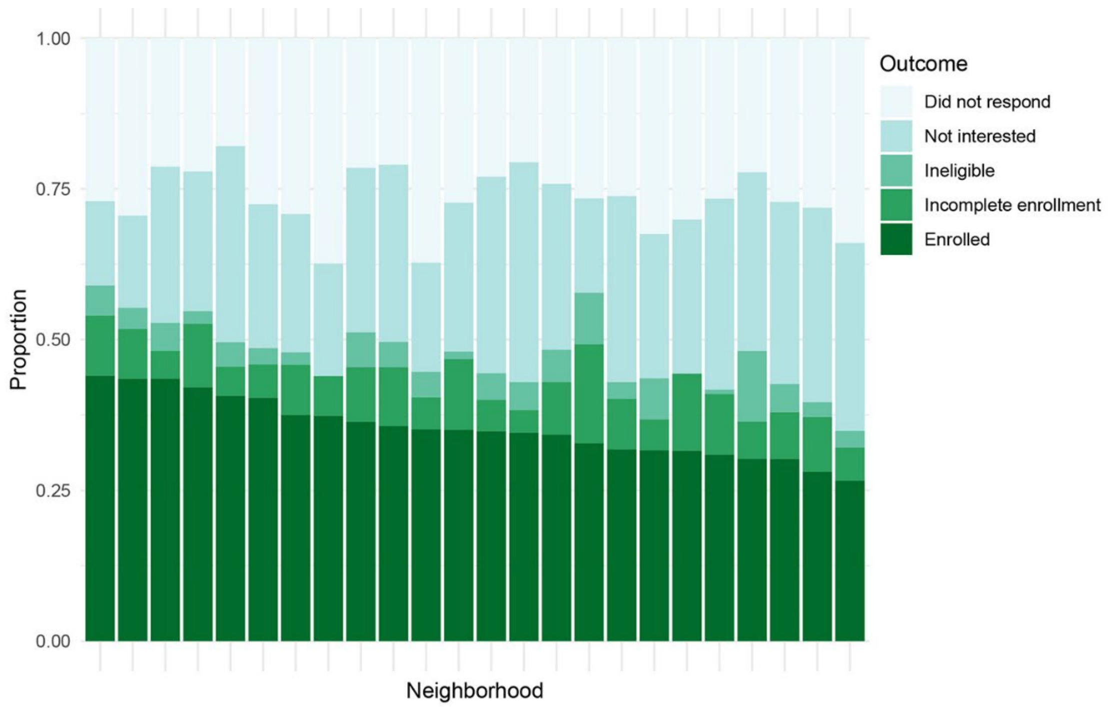

Appendix Figure 1. Proportion of households in each residential neighborhood with specified recruitment outcomes in study of tick-control interventions, New York. Bars represent each of the 24 neighborhoods in order of declining participation. Households were saturated with contacts, including mailings, door-tags, in-person visits, and, when phone numbers were available, phone calls.

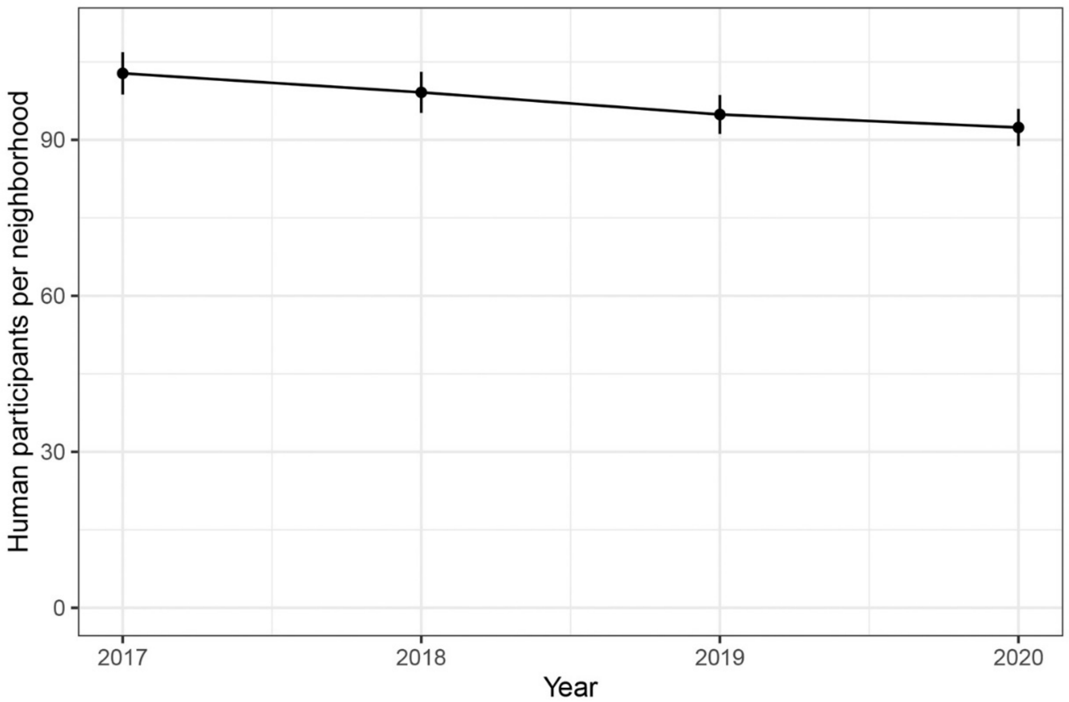

Appendix Figure 2. Mean number of human participants enrolled in study of tick-control interventions in each residential neighborhood, New York, 2017–2020. Error bars represent standard errors.

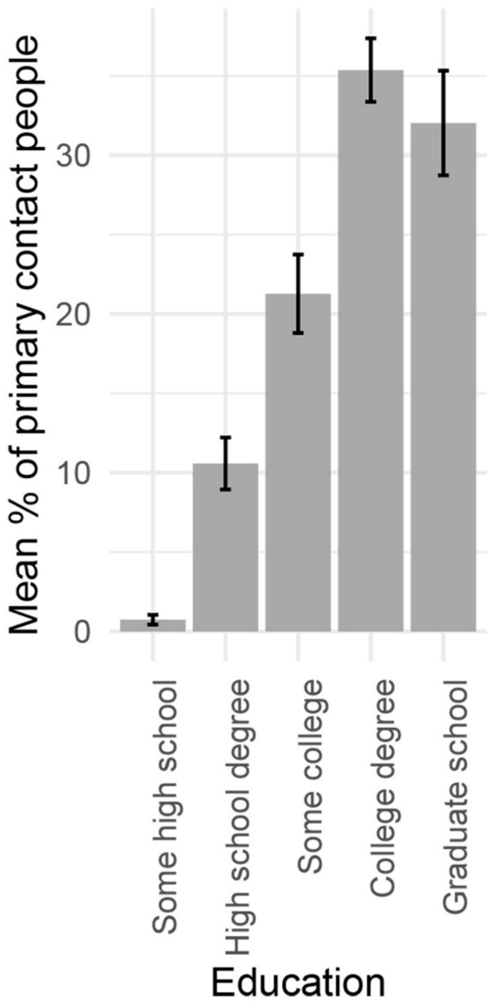

Appendix Figure 3. Mean percentage of primary contact people in each residential New York neighborhood participating in study of tick-control interventions who had the indicated level of educational attainment, averaged across the 24 neighborhoods. Error bars represent standard errors.

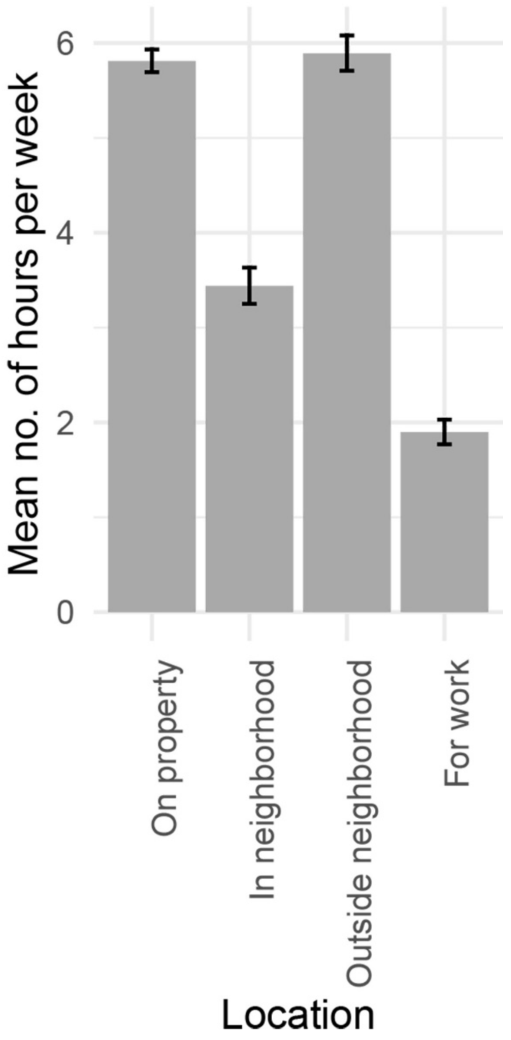

Appendix Figure 4. Mean number of hours spent outside per week by tick-control study participants in locations in or away from the neighborhood, based on responses to the enrollment survey administered in 2016–2017. Error bars represent standard errors.
